# Detection of pathogenic bacteria in the blood from sepsis patients using 16S rRNA gene amplicon sequencing analysis

**DOI:** 10.1371/journal.pone.0202049

**Published:** 2018-08-15

**Authors:** Nobuo Watanabe, Kirill Kryukov, So Nakagawa, Junko S. Takeuchi, Meiko Takeshita, Yukiko Kirimura, Satomi Mitsuhashi, Toru Ishihara, Hiromichi Aoki, Sadaki Inokuchi, Tadashi Imanishi, Shigeaki Inoue

**Affiliations:** 1 Department of Emergency and Critical Care Medicine, Tokai University School of Medicine, 143 Shimokasuya, Isehara, Kanagawa, Japan; 2 Department of Molecular Life Science, Tokai University School of Medicine, 143 Shimokasuya, Isehara, Kanagawa, Japan; 3 Micro/Nano Technology Center, Tokai University, 411 Kitakaname, Hiratsuka, Kanagawa, Japan; 4 Department of General Medicine, Tokai University School of Medicine, 143 Shimokasuya, Isehara, Kanagawa, Japan; Mayo Clinic Arizona, UNITED STATES

## Abstract

Prompt identification of causative pathogenic bacteria is imperative for the treatment of patients suffering from infectious diseases, including sepsis and pneumonia. However, current culture-based methodologies have several drawbacks including their limitation of use to culturable bacterial species. To circumvent these problems, we attempted to detect bacterial DNA in blood using next-generation DNA sequencing (NGS) technology. We conducted metagenomic and 16S ribosomal RNA (rRNA) gene amplicon sequencing of DNA extracted from bacteria-spiked blood using an Ion Personal Genome Machine. NGS data was analyzed using our in-house pipeline Genome Search Toolkit and database GenomeSync. The metagenomic sequencing analysis successfully detected three gram-positive and three gram-negative bacteria spiked in the blood, which was associated with a significant portion of non-bacterial reads, even though human blood cells were separated by low-speed centrifugation prior to DNA extraction. Sequencing analysis of seven variable regions of the 16S rRNA gene amplicon also successfully detected all six bacteria spiked in the blood. The methodology using 16S rRNA gene amplicon analysis was verified using DNA from the blood of six patients with sepsis and four healthy volunteers with potential pathogenic bacteria in the blood being identified at the species level. These findings suggest that our system will be a potential platform for practical diagnosis in the future.

## Introduction

Bacterial infection is one of the leading causes of death worldwide. Prompt and accurate identification of etiological pathogenic bacteria is imperative for proper treatment of patients suffering from infectious diseases, including sepsis and pneumonia. Since the 19th century, culture-based identification methodologies have been a mainstay for the diagnosis of pathogenic bacteria in the specimens. However, culture methods have the following drawbacks: i) the culture period for some bacteria requires up to 1 week, ii) anaerobic bacteria are generally difficult to culture, and iii) poor colony formation is inevitably associated with blood from patients treated with antibiotics [1]. To circumvent these problems, we sought to detect and identify bacteria in patient specimens based on their DNA sequences using next generation DNA sequencing (NGS) technology.

NGS technology has been rapidly developing and can read more than a million DNA strands in a single run. In parallel with the development of this technology, sequence information regarding the bacterial genome has also been accumulating in publicly-available DNA databases such as GenBank, owing to a large part to initiatives such as the human microbiome project [2]. Currently, the number of genome sequences of bacterial species stored in the GenBank database exceeds one million. NGS methodology has been increasingly employed in the clarification of microbiota, especially in the gut. There are primarily two ways to detect bacterial DNA by using NGS methods. One approach is metagenome sequencing, which examines whole DNA of the microbial communities within a given sample without isolation or cultivation of individual species. The second approach is amplicon sequencing of the 16S ribosomal RNA (rRNA) gene (16S rRNA amplicon sequencing) since the 16S rRNA gene is universally encoded in all bacterial chromosomes. Recently, several groups have applied NGS methods for the detection of pathogenic bacteria in the blood of patients with infectious diseases [3–6]. However, many possible combinations are available for conducting NGS analysis, including not only various DNA extraction methods, but also different instrumentation platforms, which may generate different results. As a result, it remains unclear what methodology is most suitable and reliable for the identification of pathogens in blood samples [7,8].

In an effort to establish an NGS-based method for the diagnosis of bacterial infectious diseases, we compared the accuracy and efficiency of metagenome analysis and 16S rRNA gene amplicon analysis using Ion Personal Genome Machine (PGM) (Thermo Fisher Scientific) with DNA samples prepared from bacteria-spiked blood. The resultant bacterial DNA reads were analyzed using our in-house metagenomic data analysis pipeline Genome Search Toolkit (GSTK; http://kirill-kryukov.com/study/tools/gstk/). We also compared the detection efficiency using whole blood and plasma. We found that 16S rRNA amplicon analysis of whole blood using the GSTK suite successfully detected both gram-positive and gram-negative bacteria in the blood. The effectiveness of our protocol was verified by testing blood from patients with sepsis.

## Materials and methods

### Blood samples

The study and protocol were approved by the Clinical Ethical Committee of Tokai University Medical School (14R-220). Blood specimens were collected in heparin-coated collection tubes from informed patients who provided written consent. Four healthy volunteers who also provided written informed consent, and ten sepsis patients, three of which ultimately had positive blood cultures, participated in the study.

Stock cultures of three common gram-positive bacterial strains (*Staphylococcus aureus*, *Streptococcus pneumoniae*, and *Enterococcus faecalis*) and three common gram-negative bacterial strains (*Pseudomonas aeruginosa*, *Escherichia coli*, and *Haemophilus influenzae*) were provided by the Tokai University Hospital Microbe Analysis Laboratory. The *H*. *influenzae* culture was grown on chocolate agar plates and the remaining cultures were grown on sheep blood agar plates. Bacterial colonies on the plates were collected in PBS using sterile cotton swabs and stored at ‒80°C in 15% glycerol at an optical density (OD) based on 600 nm-scattering ranging from 0.2–2.4 OD_600_ units. On the day of the experiments, the bacterial stocks were thawed and a mixture of the three gram-positive or three gram-negative bacteria was added to freshly drawn blood at a 1:100 ratio. Total DNA was extracted from the bacteria-spiked blood as described below. In some experiments, the bacteria-spiked blood was centrifuged by low speed centrifugation at various gravitational forces (20 × g–960 × g) with an angle rotor for 10 min at 4°C to obtain plasma. The bacteria in the plasma fractions were subsequently sedimented by high speed centrifugation at 6,000 × g for 20 min.

### DNA isolation

Total DNA in blood or plasma was isolated using a DNeasy Blood & Tissue Kit (Qiagen, Hilden, Germany) according to the manufacture’s instruction with some modifications for the co-isolation of bacterial DNA. The protocol is available at dx.doi.org/10.17504/protocols.io.qqidvue. Typically, 10 μl of lysozyme (200 mg/ml) and 5 μl of 20% Triton X-100 were added to a 100 μl sample of blood or plasma. After 30-min incubation at 37°C, proteinase K, which was supplied with the kit, was added to the samples and the total volume was adjusted to 220 μl. The samples were combined with 4 μl of RNase A (100 mg/ml) and incubated at room temperature for 2 min before adding 200 μl of Buffer AL and incubated at 56°C for 10 min. The subsequent isolation procedures were carried out exactly according to the instructions of the manufacturer. DNA concentrations were determined using the Qubit fluorometric quantitation method according to the manufacture’s instruction (Thermo Fisher Scientific).

### Real-time PCR analysis

Full-length bacterial 16S rRNA genes were quantified using real-time PCR and an Applied Biosystems 7700 sequencer using the KOD-SYBR Green method (TOYOBO, Tokyo, Japan). The following primer set was used: 16S rRNA-Forward (27F), 5´–AGAGTTTGATCCTGGCTCAG–3´; and 16S-rRNA-Reverse (1492R), 5´–GGCTACCTTGTTACGACTT –3´. The cycling conditions were as follows: Stage 1, 95°C for 20 sec; Stage 2, 40 consecutive cycles of 95°C for 3 sec, 55°C for 10 sec, and 70°C for 60 sec; Stage 3, 95°C for 15 sec, 60°C for 60 sec, and 95°C for 15 sec.

### Direct DNA sequencing (shotgun metagenome analysis) using Ion PGM

Approximately 100 ng of total DNA from blood or plasma samples was sheared into approximately 400-bp fragments by focused ultra-sonication (Covaris M220) according to the manufacture’s protocol. The DNA fragments of the appropriate size range were separated using the E-gel electrophoresis system (Thermo Fisher Scientific) according to manufacturer’s instructions. A DNA library was prepared using an Ion Plus Fragment Library kit and Ion Xpress Barcode Adaptors (Thermo Fisher Scientific). DNA concentrations of the samples, both before and after library preparation, were determined using a Bioanalyzer (Agilent). The Barcode-label concentration for each library sample was adjusted to 26 pM and 16 library samples were mixed at a concentration 130 atto-moles each, followed by amplification by emersion PCR and sequencing on Ion PGM with a 316 Chip.

### 16S rRNA amplicon sequencing with Ion PGM

Seven variable regions of the 16S rRNA gene in the DNA samples were amplified by PCR using an Ion 16S™ Metagenomics Kit (Thermo Fisher Scientific) as previously described [8] with the following modifications. Equal volumes of elution fractions from the DNA isolation column were separately amplified using the first set of primers (V2, V4, and V8) or the second set primers (V3, V6-7, and V9) with 25 cycles. The two amplicons were combined and the library was prepared. A procedure subsequent to the Ion PGM analysis was performed for shotgun sequencing, as described. For the clinical samples, unless otherwise indicated, approximately 30 ng of blood DNA was amplified using the initial PCR with 30 cycles.

### NGS data analysis

To identify the bacterial species and microbial composition in a given sample, we analyzed the NGS read data using the Ion Reporter with default parameters (Thermo Fisher Scientific) or the GSTK software suite. The Greengenes [9] and GenomeSync (http://genomesync.org) databases were used for the Ion Reporter and GSTK analyses, respectively. For the homology search, the National Center for Biotechnology Information (NCBI) Basic Local Alignment Search Tool N (BLASTN) was used for both analyses, although the parameters were different (unknown for Ion Reporter and “-evalue 3.80e-2 -dbsize 3200000000” for GSTK). All the NGS data are available from the DDBJ DRA database (https://www.ddbj.nig.ac.jp/dra/index-e.html) under accession numbers DRR138887 to DRR138913.

## Results and discussion

### Enrichment of bacteria in the blood samples

For the metagenome analysis of bacteria in the blood, minimizing the proportion of human-derived DNA is important. To this end, we first assessed conditions in which to enrich the bacteria in the blood. Control blood was inoculated with a pool of gram-positive bacteria (*Staphylococcus aureus*, *Streptococcus pneumoniae*, and *Enterococcus faecalis*) or gram-negative bacteria (*Pseudomonas aeruginosa*, *Escherichia coli*, and *Haemophilus influenzae*) and subsequently centrifuged at 3 × g, 20 × g, 100 × g, or 960 × g for 10 min. While each of these centrifugation conditions separated the blood samples into the particulate fraction (erythrocytes and white blood cells) and plasma fraction, the volume of the resulting plasma increased when higher centrifugal forces were used. As an index of bacterial counts, the 16S rRNA gene in the plasma was quantitated using real-time PCR. It was revealed that a significant amount of both gram-positive and -negative bacteria remained in the plasma after centrifugation at 3 × g or 20 × g, but at 100 × g and above, the recovery of DNA from both types of bacteria decreased (S1 Fig). Thus, with centrifugation at up to 20 × g, the bacterial cells remained suspended in the plasma while the human blood cells were eliminated.

### Metagenome sequencing analysis of whole blood and its 20 × g clarified plasma

**Fig 1:**
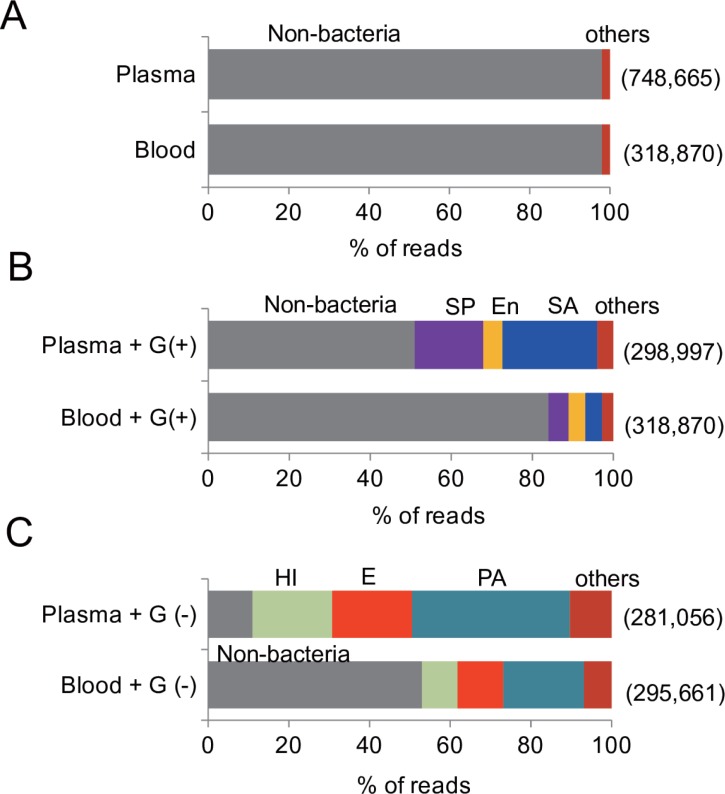
Bacterial composition of whole blood and its 20 × g plasma evaluated using metagenome sequencing and GSTK analysis. DNA was extracted from control blood (A), gram-positive bacteria-spiked blood (B), gram-negative bacteria-spiked blood (C), or their respective plasma after clarification by 20 × g centrifugation. The numbers in the parenthesis to the right show the number of reads obtained from the sequencing run. The abbreviation are as follows: SA, *Staphylococcus aureus*; SP, *Streptococcus pneumoniae*; En, *Enterococcus faecalis*; PA, *Pseudomonas aeruginosa*; E, *Escherichia coli*; and HI, *Haemophilus influenzae*.

We compared the efficiency of bacterial detection between whole blood samples and samples of the plasma fraction clarified at 20 × g by analyzing the NGS reads obtained using the Ion PGM sequencer and the GSTK pipeline. In analysis of both the control blood and the plasma, more than 95% of the NGS reads were dominated by non-bacterial sequences, probably derived from contaminating human DNA (Fig 1A). Supplementation of gram-positive bacteria to the samples (Fig 1B) resulted in the detection of all the three bacterial DNAs and their identification at the species level in both the whole blood samples and in the 20 × g plasma samples. Consistent with our assumption, with the exception of *En*. *faecalis*, the frequency of each bacterial read number was greater than 3-fold in abundance in the 20 × g plasma compared with the whole blood samples. Similarly, analysis of the NGS data resulted in the detection of all the three gram-negative bacteria that were inoculated in the blood, with their abundance being twice as high in the 20 × g plasma samples than in whole blood samples (Fig 1C). These results demonstrated that the elimination of blood cells by centrifugation is advantageous for the detection of bacteria in blood using direct DNA sequencing analysis, and that our protocol was reliable for detecting both gram-positive and gram-negative bacteria in blood.

### Analysis of the 16S rRNA amplicon sequencing data

**Fig 2:**
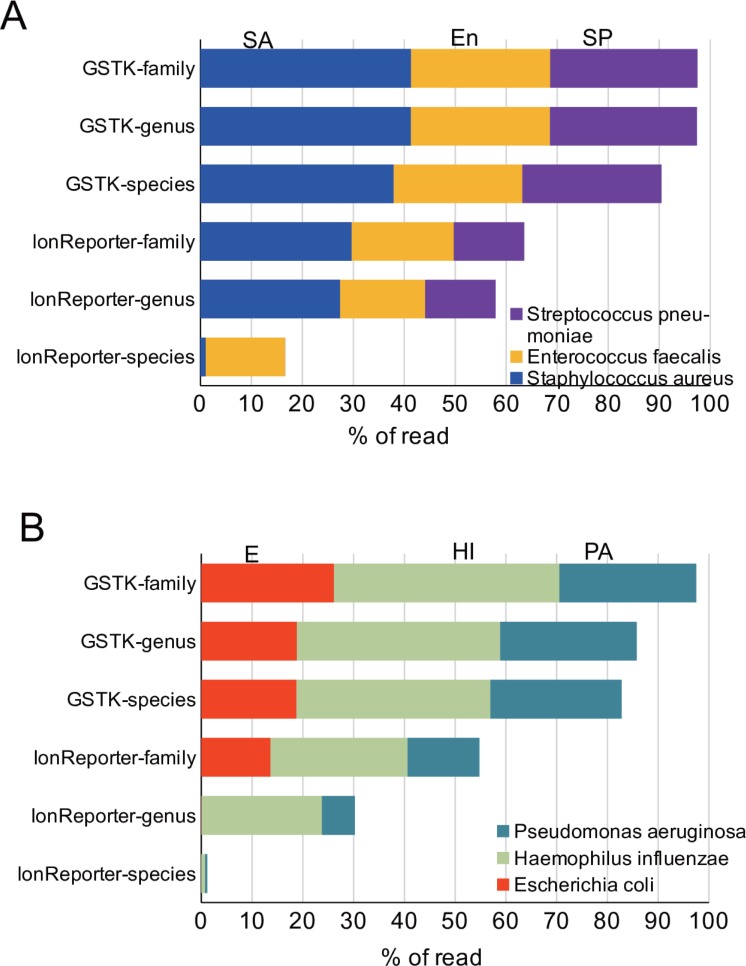
Bacterial composition identified by 16S rRNA gene amplicon sequencing followed by GSTK or Ion Reporter analysis of control and bacteria-spiked blood. Seven variable regions of the 16S rRNA gene were amplified by PCR from either gram-positive bacteria-spiked whole blood (A) or gram-negative bacteria-spiked whole blood (B) used in Fig 1, and sequenced using Ion PGM. The sequence reads were analyzed using GSTK or Ion Reporter suite software.

Since the results from the metagenome analysis of the 20 × g plasma specimens included a large number of non-bacterial reads, possibly derived not only from leukocyte contamination and cell-free human DNA [10], we compared the metagenome shotgun results with amplicon sequencing of the variable regions of the 16S rRNA gene, which in principle was expected to exclude human DNA. As a result, we found that control blood still contained bacterial DNA reads that were not derived from the added bacteria, although the number of total reads of this library was less than 1% of those from either group of bacteria-supplemented blood (3,315 for the control samples, 490,179 for the gram-positive samples, and 514,679 for the gram-negative samples). The primary bacterial species detected in the control blood were *Rudaea cellulosilytica* (6.7%), *E*. *coli* (4.8%), and *Luteibacter* sp. (2.7%). These bacteria may have contaminated the specimens during the experimental processes, including at the time of blood collection. In contrast, in the blood supplemented with the gram-positive or gram-negative bacteria, more than 80% of reads were annotated to the species level of the three respective bacteria used to spike the samples (Fig 2A and 2B). At the family level of classification, more than 95% of the reads were assigned to the respective families of the added bacteria.

In addition to the GSTK analysis, we also used the Ion Reporter software, which is the recommended suite of analytical programs for analysis of Ion PGM sequence reads. This allowed us to compare the two methods and to evaluate their accuracy. The results indicated that GSTK was more accurate at identifying the bacterial at both the species and genus levels, and to a lesser extent at the family level. This may have been because of differences in the databases used for the BLAST search as well as the parameters set for the BLASTN search (see Materials and Methods). Thus, the results demonstrated that the analysis of the 16S rRNA amplicon using the GSTK pipeline successfully detected the gram-positive and gram-negative bacterial species in the blood samples.

### Optimization of the 16S rRNA amplicon analysis of clinical samples

We assessed the practical applicability of our 16S rRNA amplicon analysis methodology using clinical samples. Blood from sepsis patients was analyzed as we sought to optimize the conditions used in the process for the initial amplification of the variable region by PCR. Preliminary experiments using 30 ng blood DNA as input template revealed that 25 cycles were insufficient to consistently obtain adequate amounts of variable region amplicons—a total amount of 30 ng of variable region amplicon is required for the subsequent conjugation reaction with adaptors and barcodes in NGS library preparation with the Ion 16S metagenomics kit. Therefore, the PCR cycling profile was increased to 30 cycles of amplification. Subsequently, the effect of the amount of input blood DNA for the initial PCR was also assessed. Increasing the input DNA to more than 30 ng resulted in not only an increase in the amount of variable region amplicons, but also in the appearance of longer amplification products of 400 to 700 bp, especially with primer set 2. It was unlikely that these longer products were derived from variable regions (V3, V6–7, V9) being amplified (S2A Fig). GSTK analysis of the Ion PGM sequencing results also revealed that increasing the input amount of blood DNA conversely decreased the total sequence reads for the bacteria, while increasing the sequence reads related to humans DNA (S2B Fig). Thus, excess blood DNA, at least above 30 ng, negatively affected the proportion of the bacterial reads.

### Application of 16S rRNA amplicon analysis to clinical samples

**Fig 3:**
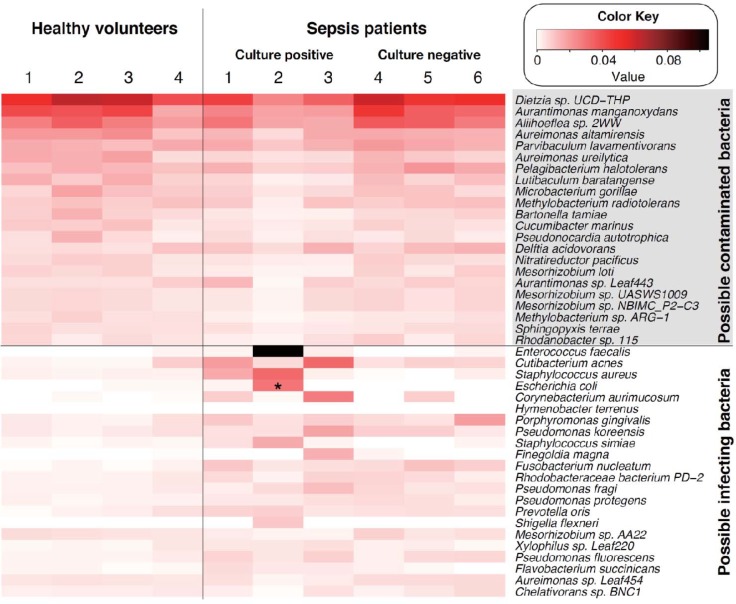
Bacterial composition in whole blood from healthy volunteers and sepsis patients. A heat map chart of possible infecting species of bacteria and possible environment-contaminated bacterial species detected in blood samples of healthy volunteers and sepsis patients. An asterisk indicates that the bacterial species was also detected by blood culture testing. Amplicons were prepared using 30 ng of input DNA for the PCR amplification. The figure was generated using a gplots module in the R software.

To evaluate the effectiveness of our entire NGS procedure, we analyzed blood DNA obtained from six sepsis patients and four healthy volunteers in a single set of experiments using a DNA input level of 30 ng for each sample (Fig 3). We found that several bacterial species were commonly detected, not only in sepsis patients, but also in the healthy volunteers, suggesting a possible environment-derived contaminating bacteria as had occurred during DNA isolation or the library preparation processes. Therefore, to discriminate the contaminating bacterial species, all bacterial species with a mean abundance > 0.5% in the healthy volunteers were aligned according to relative abundance and set aside as “possible contaminated bacterial species.” The remaining bacterial species in the samples from the sepsis patients were aligned in the order of abundance and defined as “possible infecting bacterial species.” The resultant heat map chart clearly distinguished the “possible contaminated bacteria” and the “possible infecting bacteria” detected in the blood of the sepsis patients (Fig 3). Thus, inclusion of samples derived from healthy volunteers in the same assay set may be essential in order to discern potential infecting bacteria from otherwise undistinguishable arrays of bacterial species.

Among the sepsis patients analyzed, patients 1, 2 and 3 had positive blood culture results. It is of note that, although patient 2 was positive for only *E*. *coli* in the blood culture test, our NGS method, in addition to detecting *E*. *coli* (2.9%), also detected *En*. *faecalis* (10.5%) and *Staphylococcus aureus* (3.3%) in the top three places (Fig 3). Since the cause of sepsis in this patient was the accidental puncture of the large intestine, detection of *En*. *faecalis* was reasonable and suggested that this patient likely contracted enterobacterium-triggered poly-microbial sepsis. The results clearly demonstrated that our NGS method was able to detect not only culture-positive bacteria, but also bacteria that was undetectable by culture methods. On the other hand, patient 1 was positive for *Staphylococcus epidermidis* according to the blood culture test, but its abundancy was only 0.18% according to the NGS analysis and its order in prevalence was number 93. The elusiveness in detecting some culture-positive bacteria by NGS may be attributable to differences in the amplification period of the blood culture. In routine clinical testing at our hospital facility, blood samples are continuously cultured up to 1 week unless the turbidity of the medium due to bacterial growth reaches a certain level. That is, the NGS results represent the bacteremia status at the time of blood collection, whereas results from the blood culture test may include over-represented results due to the *ex vivo* culturing. Although all patients were negative for *Cutibacterium (Propionibacterium) acne* in the blood test, this opportunistic bacterial species was detected by the NGS method at a relatively abundant level among the patients that had a positive blood culture test. This result may provide intriguing insight for sepsis research in the future.

NGS has been gaining attention as a potential diagnostic tool to detect bacteria in specimens of various infectious diseases. Regarding blood samples, four groups have reported the application of NGS to the diagnosis of sepsis [3–6]. Two of research groups conducted direct DNA sequencing analysis of cell-free DNA in plasma [3,4]. However, both groups also faced a significant issue of sequence reads derived from contaminating human cells, which accounts for as much as 98% of the total reads, downgrading the proportion of pathogen-derived reads from the blood specimens. Gosiewski et al. [5] and Gyarmati et al. [6] also conducted 16S rRNA gene amplicon analyses; however, neither of these studies could compellingly identify the causative bacteria at the species level. One potential reason for the lack of conclusiveness may be attributable to an insufficiency in reads information since they analyzed only V1–V3 or V3–V4 region amplicon. In the current study, we sequenced seven variable regions and analyzed the results using the GSTK program, and successfully detected all three gram-positive and all three gram-negative bacteria in a model blood sample. It should be emphasized that the relative abundance of bacterial reads from our 16S rRNA gene amplicon sequencing analysis was comparable to that detected by our metagenome sequencing analysis. Using the 16S rRNA amplicon analysis of blood from patients with sepsis, we were able to detect potential causative bacteria, including both bacteria identified by culture methods as well as bacteria that were not detected by culture. Although the sample number in this pilot study was limited, the success of our approach has motivated us to continue applying this methodology. With the accumulation of patient data over time, the superior aspects as well as the drawbacks of our methodology will be revealed and guide further improvements in future.

While our method produced satisfactory results in terms of its accuracy, it still has several hurdles that must be overcome before employing it in clinical use. One of these is the turnaround time; currently nearly 1 week is required to complete entire process from library preparation to data analysis using the GSTK program. However, routine blood culture sometimes requires a similar amount of time. Reducing the cost is also important for routine use of the NGS method; the cost per sample of IonPGM analysis is $100, which is three times higher than that of a blood culture test. Other challenges include increasing the sensitivity and quantitative nature of the results. Differences in the DNA extraction efficiency for individual bacteria, copy number of 16S rRNA gene, and the amplification efficiency of different regions may all affect the outcome [11,12]. Further, the detection of genes responsible for antibiotic resistance is an important issue in NGS-based diagnosis of sepsis. Further studies on these issues are in progress in order to refine our methodology to a level applicable for its diagnostic use in the future.

## Conclusions

The present NGS-based 16S rRNA amplicon sequencing combined with GSTK analysis could be a useful diagnostic tool for the determination of pathogenic bacteria in blood and may prove to be a potential platform for clinical applications in the future.

## Supporting information

S1 Fig Effect of centrifugal force on the separation of bacteria in the blood. A mixture of three gram-negative or gram-positive bacteria was added to the blood from healthy volunteers and centrifuged at the indicated force. The DNA contents in the resulting plasma were quantitated using real-time PCR. The data are representative results obtained from a single assay.
(EPS)

S2 Fig Optimization of conditions for 16S rRNA gene amplicon analysis of blood DNA isolated from sepsis patients. (A) Agarose gel electrophoresis analysis of 16S rRNA gene V region amplicons obtained using primer set 1 and set 2. The amplicons were generated with the indicated amount of blood DNA input from a sepsis patient by PCR using 30 cycles of amplification. The blue segments indicate that the expected size of the V region amplicons (150–300 bp). (B) Effects of the quantity of input DNA on the prevalence of read for *Homo sapiens*. The 16S rRNA gene amplicons were generated either at 30 ng input or larger amount as indicated from four sepsis patients and analyzed using Ion PGM and GSTK suite software.
(EPS)
